# Scale-up and evaluation of high solid ionic liquid pretreatment and enzymatic hydrolysis of switchgrass

**DOI:** 10.1186/1754-6834-6-154

**Published:** 2013-10-25

**Authors:** Chenlin Li, Deepti Tanjore, Wei He, Jessica Wong, James L Gardner, Kenneth L Sale, Blake A Simmons, Seema Singh

**Affiliations:** 1 Advanced Biofuels Process Demonstration Unit, Lawrence Berkeley National Laboratory, Emeryville, CA, USA; 2 Deconstruction Division, Joint BioEnergy Institute, Emeryville, CA, USA; 3 Biological and Materials Science Center, Sandia National Laboratories, Livermore, CA, USA

**Keywords:** Scale-up, Pretreatment, Saccharification, Ionic liquid, High solid loading, Viscosity, Inhibition

## Abstract

**Background:**

Ionic liquid (IL) pretreatment is receiving significant attention as a potential process that enables fractionation of lignocellulosic biomass and produces high yields of fermentable sugars suitable for the production of renewable fuels. However, successful optimization and scale up of IL pretreatment involves challenges, such as high solids loading, biomass handling and transfer, washing of pretreated solids and formation of inhibitors, which are not addressed during the development stages at the small scale in a laboratory environment. As a first in the research community, the Joint BioEnergy Institute, in collaboration with the Advanced Biofuels Process Demonstration Unit, a Department of Energy funded facility that supports academic and industrial entities in scaling their novel biofuels enabling technologies, have performed benchmark studies to identify key challenges associated with IL pretreatment using 1-ethyl-3-methylimidazolium acetate and subsequent enzymatic saccharification beyond bench scale.

**Results:**

Using switchgrass as the model feedstock, we have successfully executed 600-fold, relative to the bench scale (6 L vs 0.01 L), scale-up of IL pretreatment at 15% (w/w) biomass loading. Results show that IL pretreatment at 15% biomass generates a product containing 87.5% of glucan, 42.6% of xylan and only 22.8% of lignin relative to the starting material. The pretreated biomass is efficiently converted into monosaccharides during subsequent enzymatic hydrolysis at 10% loading over a 150-fold scale of operations (1.5 L vs 0.01 L) with 99.8% fermentable sugar conversion. The yield of glucose and xylose in the liquid streams were 94.8% and 62.2%, respectively, and the hydrolysate generated contains high titers of fermentable sugars (62.1 g/L of glucose and 5.4 g/L cellobiose). The overall glucan and xylan balance from pretreatment and saccharification were 95.0% and 77.1%, respectively. Enzymatic inhibition by [C_2_mim][OAc] at high solids loadings requires further process optimization to obtain higher yields of fermentable sugars.

**Conclusion:**

Results from this initial scale up evaluation indicate that the IL-based conversion technology can be effectively scaled to larger operations and the current study establishes the first scaling parameters for this conversion pathway but several issues must be addressed before a commercially viable technology can be realized, most notably reduction in water consumption and efficient IL recycle.

## Background

The state of technology for the conversion of agricultural residues, perennial grasses, woody perennials and forest products for the production of biofuels is rapidly advancing [1,2]. Production of clean fermentable sugars for biofuel production requires pretreating the biomass to overcome the recalcitrance of lignocellulose and render the polysaccharides within the plant cell walls amenable to enzymatic saccharification [2-5]. Among the leading pretreatment technologies, certain ionic liquids (ILs) have recently been shown to efficiently fractionate biomass and provide clean sugar substrate for the production of ethanol and other advanced biofuels [6-11].

Previous work has illustrated several favorable properties of IL pretreatment for biomass deconstruction at the laboratory scale. These include efficient biomass dissolution and disruption, reduced cellulose crystallinity and lignin content in the recovered product, enhanced biomass saccharification, and low toxicity and environmental impact [7,9-15]. However, most of the IL pretreatment data to date were obtained at low solid loading (3-10%) and at the 10 to 50 mL level of operation [16-18], which cannot be directly translated to industrially relevant scales. Thus, liter-scale experiments are a necessary intermediate step between bench- and pilot-scale in order to identify operational parameters and potential problems associated with scale-up prior to pilot-scale and full-scale commercial operations. This is especially true as IL pretreatment is a relatively new pretreatment technology and no scale-up systems have been described in the scientific literature.

The advantages of using high-solid loadings (≥15%) in the unit operations of lignocellulose conversion include increased sugar and ethanol concentrations and decreased production and capital cost [4]. However using high-solids in the IL process at large-scale is still relatively unexplored, and more research is required to overcome certain challenges, including high quantity materials handling, equipment mass transfer limitations, rheological problems, and solvent usage for washing, that are not as apparent at low solids loadings. In addition, high solid enzymatic saccharification has been suggested to increase the initial conversion rate and final fermentable sugar concentrations [19], but can exacerbate enzyme inhibition and pose rheological challenges that must be taken into account. Cellulase and hemicellulase inhibitors include products such as glucose and xylose, intermediates such as cellobiose, degradation products arising from pretreatment, solvents such as IL and ethanol (the latter used for precipitation or washing, as well as lignin due to non-specific binding and solubilized phenolics) [20-23]. Detoxification of lignocellulosic hydrolysates via biological, chemical and physical conditioning processes have been used to remove inhibitors prior to or after enzymatic hydrolysis [23,24]. For IL pretreatment, post-washing of recovered materials with water or other solvents to dilute the IL to non-inhibitory levels and to remove other biomass-derived products has been investigated [20,22]. Other options include developing IL-tolerant enzymes and microorganisms to conduct single pot configuration for enzyme hydrolysis and microbial fermentation [25,26], or using lower IL concentration (20-50%, w/v) in water to pretreat biomass and potentially reduce the amount of washing required prior to enzymatic saccharification [21,27]. To date, all these potential alternatives have been limited to the lab-scale level of development and require more investigation before scale-up can occur.

As a first in the research community, the Joint BioEnergy Institute (JBEI), in collaboration with the Advanced Biofuels Process Demonstration Unit (ABPDU), a Department of Energy (DOE)-funded facility that supports academic and industrial entities in scaling their novel technologies, have performed benchmark studies to identify the key large-scale process issues associated with IL pretreatment and subsequent saccharification. Building on the small scale optimization data, the scope of the current study encompasses the following aims relevant to assessing IL pretreatment for biofuel production: 1) evaluating IL pretreatment and subsequent enzymatic saccharification for high-solids loading at liter scales; 2) understanding the rheological properties of IL pretreated biomass that affect material handling during process integration; 3) identifying the critical requirements of the washing unit operation to minimize inhibition; 4) tracking the material and energy flow for product/solvent and energy recovery.

## Results and discussion

### Scale up of IL pretreatment

Certain ILs, such as 1-ethyl-3-methylimidaolzium acetate ([C_2_mim][OAc]), can dissolve a wide range of feedstocks at solids loadings less than 5% (w/w) ([9,16]. However, the large amounts of relatively expensive [C_2_mim][OAc]) required for effective reduction of biomass recalcitrance to improve subsequent rates and yields of enzymatic hydrolysis is a major concern [18]. Thus, higher solid loadings that require lower volumes of IL/water/solvent and smaller reactors would reduce capital and production costs and would be a significant step toward practical lignocellulosic IL pretreatment strategies [4,10,18,28].

**Figure 1:**
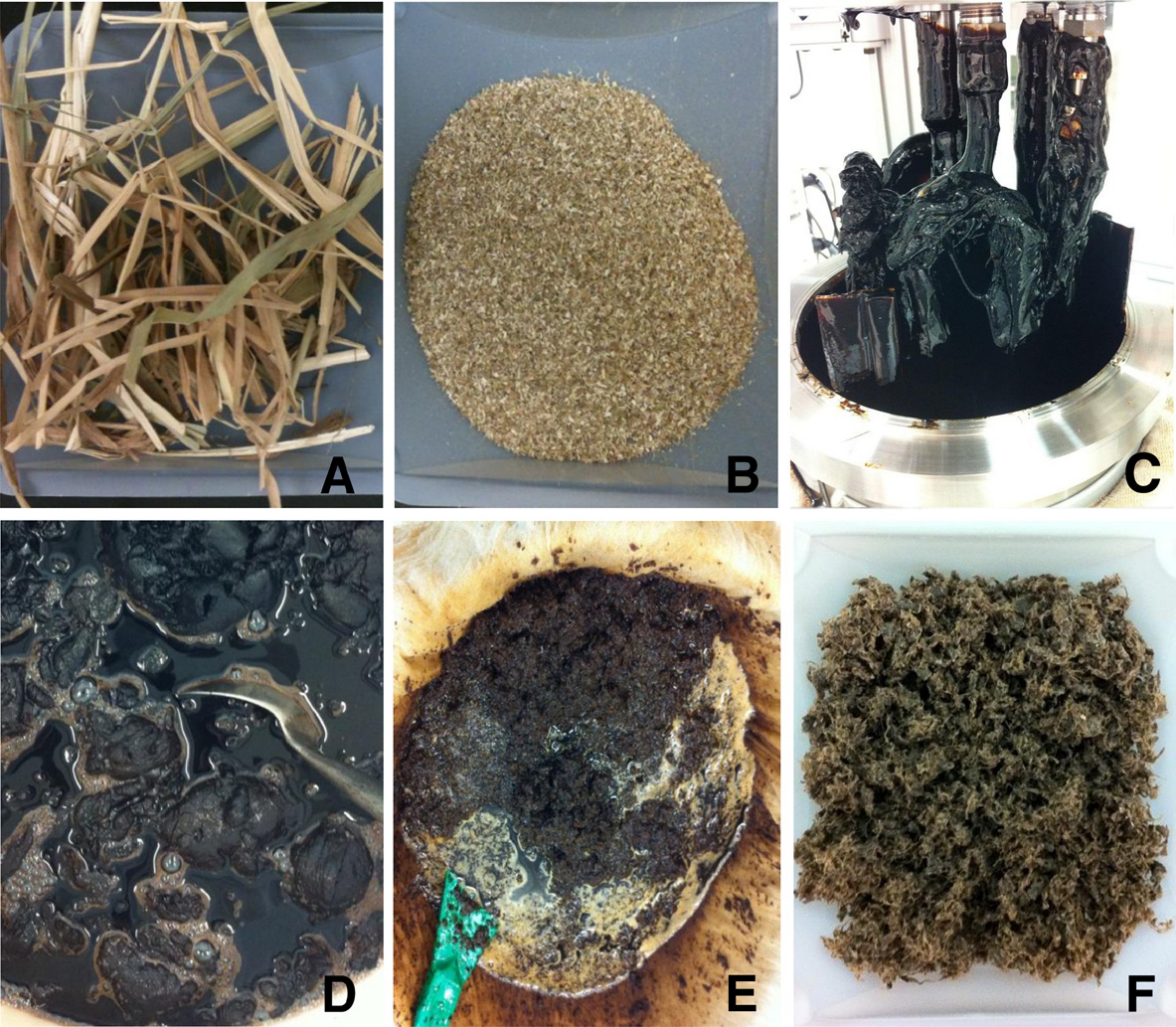
**[C**_**2**_**mim][OAc] pretreatment of switchgrass at 6 L scale.** Images depicting **(A)** switchgrass straw, **(B)** switchgrass flour after size reduction (2 mm screen), **(C)** expansion and solubilization of switchgrass in [C_2_mim][OAc] after 3 h reaction at 160°C, **(D)** gel formation upon water precipitation, **(E)** homogenized biomass undergoing washing process and **(F)** recovered switchgrass.

For the current study, 0.9 kg of milled switchgrass was mixed with [C_2_mim][OAc] and processed at 6 L scale at a solid loading of 15% (w/w) in a 10 L Parr reactor. Figure 1 presents the biomass morphologies at different stages of the IL pretreatment process. At this solid loading, switchgrass was observed to be significantly solubilized in [C_2_mim][OAc] after 3 h reaction at 160°C, which is similar to that observed at lower solid loading in 10 mL small-scale studies [15,20]. We attribute this finding to the effective and uniform mixing provided by the anchor impeller. Unlike other pretreatment methods that preserve the fibrous structure of the biomass in the slurry [4,29], the switchgrass/IL mixture has a morphology that is highly viscous with no visible signs of fibrous material remaining (Figure 1C).

Figure 1D depicts the formation of a gel phase when water is added into the [C_2_mim][OAc]-switchgrass slurry. This has been observed at biomass loadings as low as 3% (w/w) and has several consequences [20,30]. First, mixing gel phases, which are quite stiff at high solid loadings, can be problematic. Second, if the gel phase is not completely dispersed in the solvents, separation of precipitated solids from the IL is more difficult and may result in increased losses of the IL and exacerbated toxicity to downstream saccharification and fermentation processes utilizing enzymes and microorganisms [22]. Many reports in the literature on IL pretreatment utilize low biomass loadings of 3-5% and large quantities of precipitating solvent or use mixtures of acetone and alcohol for precipitation and washing [7,9,11,20]. These practices minimize the formation of the gel phase but increase solvent consumption and other processing costs. In light of these observations, we chose water as the precipitating and main washing solvent due to its cost and relative ease of use. The gel materials were homogenized with a laboratory blender to mechanically break down the gel and facilitate complete dispersion in water.

The efficient recovery of pretreated materials is an important unit operation in all pretreatment techniques. Similarly, the efficient recovery of pretreated biomass and removal of residual solutes is a key step in the scaled-up IL process. In this study, after precipitation and homogenization, the biomass went through 3× water, 1× ethanol, 2× ethanol/water and 1 final water washing steps (Figure 1E) to obtain clean pretreated switchgrass that is readily saccharified to fermentable sugars. After precipitation, extensive washing and filtration, the wet recovered switchgrass displays a porous structure (Figure 1F). Small-scale studies commonly use dried pretreated materials for physiochemical characterization and low loading levels for saccharification [6,11,15,16]. Drying might greatly change the characteristics of cellulosic materials. For example, air drying reduces substrate reactivity due to collapse of the supramolecular structure, whereas freeze drying better preserves the substrate morphology but still decreases the substrate accessibility compared to the samples that were never dried [29]. Enzymatic hydrolysis of cellulose requires hydrated conditions and in practice drying of materials may not be desired in a biorefinery that continuously requires substrate for fermentation. Thus, a unit operation for material drying was not employed in this process development and all pretreated materials were kept wet and stored at 4°C before enzymatic saccharification.

### Viscosity of [C_2_mim][OAc] pretreated materials

**Figure 2:**
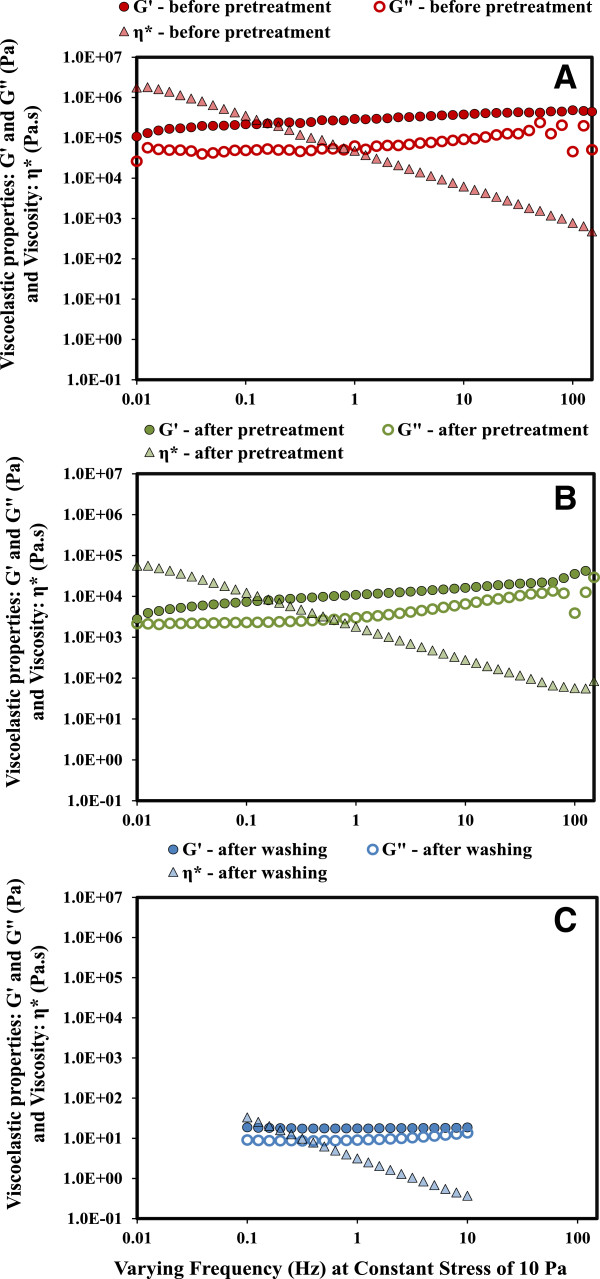
**Rheological characterization of switchgrass slurry at various stages of the pretreatment unit process (A) switchgrass/[C**_**2**_**mim][OAc] mixture before pretreatment, (B) switchgrass/[C**_**2**_**mim][OAc] mixture after pretreatment, and (C) switchgrass/water mixture after washing.**

At high solids, lignocellulosic biomass is typically fibrous and hygroscopic and requires special mixing and handling techniques at large-scale. Rheology of biomass can significantly influence the progress of chemical and biological conversion of biomass to monomeric sugars [31]. Rheological measurements have been conducted to investigate the behavior of dilute acid pretreated softwood [32] and corn stover [31,33], but very limited studies have been devoted to the rheological characteristics of IL pretreated lignocellulosic feedstocks [28]. In this study, we measured the rheological properties of the switchgrass slurry before pretreatment (15% w/w insoluble solids in [C_2_mim][OAc]), after pretreatment (15% w/w insoluble solids in [C_2_mim][OAc]), and after washing (12% w/w insoluble solids in water). The elastic (or storage) moduli (G’, Pa), viscous (or loss) moduli (G”, Pa), and complex viscosities (η*, Pa · s) within a frequency range of 0.1 to 100 Hz at a constant stress of 10 Pa were measured and shown in Figure 2. The three viscoelastic parameters, η* (10^4^ to 10^2^ Pa · s), G’ (5 × 10^4^ Pa), and G” (5 × 10^3^ Pa), obtained from switchgrass/[C_2_mim][OAc] mixtures after pretreatment were similar to those reported for dilute acid pretreated corn stover (20% w/w insoluble solids) [31], indicating that both pretreatment technologies have a similar influence on the rheological properties of biomass. These results are interesting because [C_2_mim][OAc] has a much higher viscosity (9.3 × 10^-2^ Pa · s) than water (1 × 10^-3^ Pa · s) at 25°C. The high viscosity of [C_2_mim][OAc] didn’t increase the viscosity of biomass-ionic liquid mixture and possibly led to a reduced requirement of mixing due to improved solvation of biomass. A recent study reported rheological properties of [C_2_mim][OAc] pretreated switchgrass at the 10 mL scale, where biomass was only pre-mixed with [C_2_mim][OAc] prior to the pretreatment but not throughout the process [28]. Interestingly, the shear thinning behavior and viscoelastic properties of the pretreated biomass from this 10 mL scale were similar to the results obtained from this study at 6 L scale (Figure 2), where a Parr reactor system with continuously stirring at 50 rpm was used.

To further evaluate the requirements of mixing and handling techniques for high solid loadings, it is critical to obtain a fundamental understanding of the biomass flow properties under various unit operations. In this study, we observed that the η* (5 × 10^5^ to 10^3^ Pa · s) of switchgrass/IL slurry before pretreatment was almost 100-fold higher than that after pretreatment (Figure 2A and 2B). Washing effectively removed [C_2_mim][OAc] and lignin, and also led to a significant drop in η* (5 × 10^1^ to 10^-1^ Pa · s) (Figure 2C). This continuous decrease in viscosity from before pretreatment to after pretreatment and eventually after washing indicates that the unit operations of IL pretreatment and washing are capable of making biomass more “fluid-like” and material handling more amenable in large-scale processes.

### Solids recovery and changes of chemical composition

Based on the results of 6 L scale runs, 55.3% of the starting material was recovered as solids after pretreatment with [C_2_mim][OAc]. This amount of solids recovered at the 6 L scale is similar to previous value of 49.3% for switchgrasss pretreated at 3% (w/w) loading at the 10 mL scale [16], suggesting that solids recovery is comparable with scaling to higher biomass loadings and larger volumes. The slight differences observed are likely attributed to the solid loading, mixing and cellulose regeneration method at the larger scale. Similarly, a recent study obtained a higher solid recovery of 64.9% from mixed feedstocks under the same reaction conditions but at 10% biomass loading at the 200 mL scale [17]. The mass loss is attributed to the solubilization of components such as xylan, lignin and other extractives during pretreatment with [C_2_mim][OAc], and it should be noted that there are processes in place to recover the solubilized sugars and are not considered lost to the overall conversion process [34,35]. Our recent lab scale study has demonstrated that the application of liquid-liquid extraction achieved over 90% glucose and xylose recovery from [C_2_mim][OAc] water mixture, whereas lignin was fractionated into streams with different molecular weight after pretreatment and saccharification, offering the possibility for lignin recovery and subsequent valorization [26,35].

**Figure 3:**
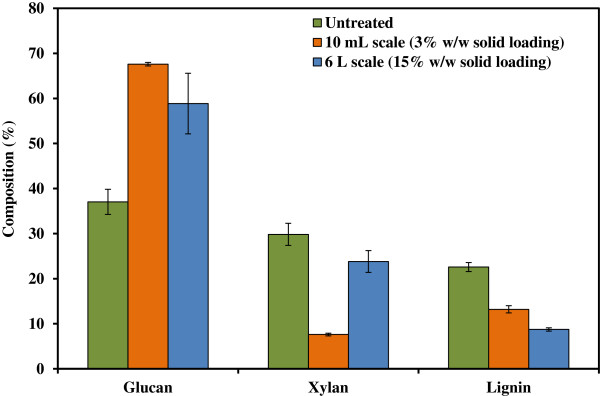
**Comparison of compositional changes in switchgrass before and after pretreatment at 10 mL and 6 L scales.** The 10 mL scale data were taken from our previous study [7].

Figure 3 illustrates the scale-up and loading effects on the changes of three major biomass components, glucan, xylan and lignin, of pretreated switchgrass in comparison to the results obtained at the 10 mL scale data from previous studies [7]. Unlike the significant removal of both xylan and lignin observed at 10 mL scale and 3% solids loading, at the 6 L scale there is significantly less lignin content (8.7% vs 13.2%) but much higher xylan content (23.8% vs 7.6%). This is likely due to the loading used in the current study, and indicates that this process configuration favorably preserves the structural carbohydrates for subsequent enzymatic saccharification and fermentation.

### Hemicellulose in the liquid fractions

**Figure 4:**
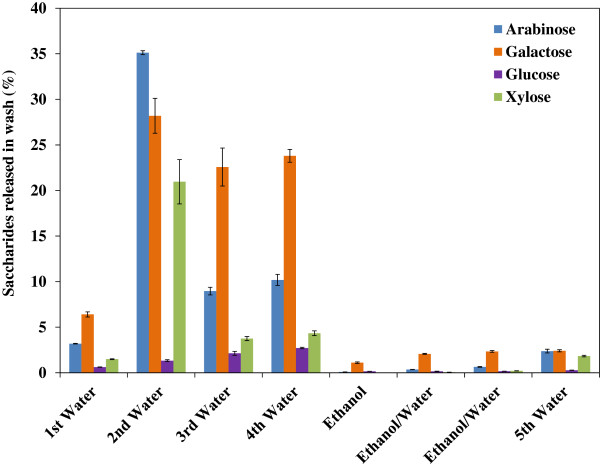
**Pattern of saccharides released (based on untreated compositions) from [C**_**2**_**mim][OAc] pretreated switchgrass into the liquid phase and its dependence on the number of washes.**

IL pretreatment can remove significant amounts of hemicellulose and small quantities of amorphous cellulose depending on the severity of reaction conditions [15], and, under some pretreatment conditions, hemicellulose can be depolymerized to oligosaccharides [7]. To understand the effect of washing on hemicellulose removal, a complete analysis on sugar released during eight washing steps was conducted using High Performance Anion Exchange Chromatography (HPAEC) after trifluoroacetic acid (TFA) digestion (Figure 4). These results show that the pattern of hemicellulose release, as measured by the xylose, arabinose and galactose contents, depends on the washing steps and the solvent used. The second wash liquor removed much higher xylose (21.4%), arabinose (34.8%) and galactose content (27.8%). This result is likely due to major fractions of three polymers still being bound to the solid in the first washing step and being released into the liquid when water was added for the 2nd wash. The third and fourth washes were also effective in removing the three sugars. The last water wash released more sugars than the previous ethanol and ethanol-water washes. This could be attributed to the ethanol wash steps (5, 6 and 7) that removed more IL and made hemicellulose less bound to the cellulose, and with the last water wash, the loosely bound hemicellulose readily dissolved into water. Small amounts of glucose were also observed in the first four water washes, which were likely released from the amorphous cellulose initially present in switchgrass. Results also show that the total loss of the initial glucan fraction in all the washing steps was 12.6% at 6 L scale, lower than the loss of 15.6% in previous small scale studies [16], further suggesting high solids loading may help retain the structural carbohydrates in the recovered solid stream.

### IL content and its impact on enzymatic saccharification

**Figure 5:**
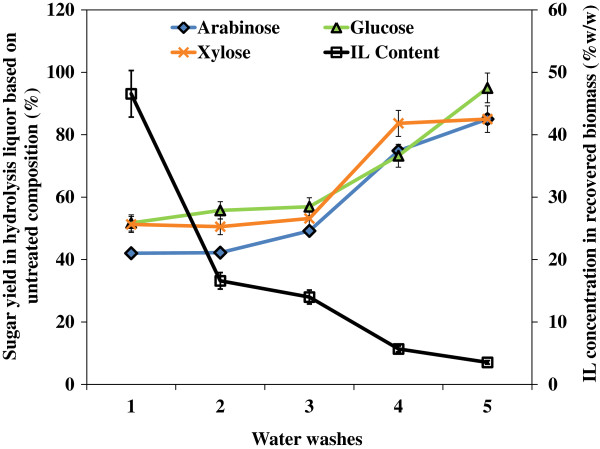
**Impact of residual [C**_**2**_**mim][OAc] in terms of saccharification inhibition.** Sugar yields were measured after saccharification (54 mg/g glucan of Cellic CTec2, 6 mg/g glucan Cellic HTec2, 50°C, pH 5.5 for 72 hours) of solids taken after each wash step. IL concentration in recovered biomass is the concentration of [C_2_mim][OAc] measured in the supernatant after complete solubilization of the solids.

Residual IL in the solids have been found to inhibit both commercial cellulases and microbial fermentation in *Saccharomyces cerevisiae*[22], requiring extensive washing after IL pretreatment. In this study, the [C_2_mim][OAc] content in both the liquid and solid fractions was measured to track the efficiency of washing steps. As shown in Table 1, the amount of [C_2_mim][OAc] in the liquid stream was significantly reduced from 43.43% (w/v) after the first water wash to 0.03% (w/v) after the final water wash. However, a certain amount of IL residue is hypothesized to stay in the solid fraction and may not be easily removed. To investigate the effects of [C_2_mim][OAc] in the solid fraction on enzymatic saccharification, a separate small-scale study was conducted to track the residual [C_2_mim][OAc] remaining in the solids after pretreatment. Samples of the solids remaining after each water washing step were taken and completely solubilized using excess concentrations of Cellic® HTec2 and CTec2 for five days to liberate the [C_2_mim][OAc]. The [C_2_mim][OAc] concentration in the liquid phase following complete solubilization was then used as a measure of the residual [C_2_mim][OAc] in the solids at each wash step. In parallel, solid samples after each wash were hydrolyzed (54 mg/g glucan of CTec2, 6 mg/g glucan HTec2, 50°C, pH 5.5) for 72 hours to determine sugar yields. Figure 5 clearly shows that the reduction of IL in the solids as a function of the number of washes yields significant increases in sugar release. At [C_2_mim][OAc] concentrations of 16.6-46.6%, all sugar yields were less than 60%, indicating the glycoside hydrolase enzymes in the commercial cocktails have reduced activity [25]. After the 4th water wash, all sugar yields in the hydrolysates reached 70-80% as the [C_2_mim][OAc] concentration dropped to 5.7%. After the final water wash, the inhibition on the sugar yields was not significant at the IL concentration of 3.5%. Both Table 1 and Figure 5 do not show significant IL removal efficiency by ethanol washing steps as indicated by the slight decrease of [C_2_mim][OAc] in liquid and solid streams. It is evident that this pretreatment configuration requires extensive washing of the biomass post-pretreatment to remove the residual [C_2_mim][OAc], which is known to inhibit downstream saccharification and fermentation [22]. The excessive use of water and waste disposal associated with washing poses challenges for the scale-up of IL pretreatment technology. Alternative options to reduce water usage have been recently reported, including the development IL-tolerant cellulase cocktails (i.e., JTherm cocktail recently developed at JBEI) that are compatible with a “single-pot” (pretreatment + saccharification), wash-free bioprocessing approach [25,26], use of lower IL concentration (25-50%, w/v) in water for biomass pretreatment that eliminates washing prior to enzymatic saccharification altogether [27,35], and the development of enzyme free, acid catalyzed IL hydrolysis process [36]. So far these alternative technologies are all limited to the lab-scale level of development and require more investigation before scale-up can occur.

**Table 1:** **Amount of [C**_**2**_**mim][OAc] present as a function of the wash process**

**Wash no.**	**Solvent**	**[C**_**2**_**mim][OAc] content (%, w/v)**
1	Water	43.43 ± 0.72
2	Water	7.56 ± 0.15
3	Water	2.06 ± 0.09
4	Water	0.48 ± 0.02
5	Ethanol	0.20 ± 0.01
6	Ethanol/water	0.12 ± 0.01
7	Ethanol/water	0.06 ± 0.00
8	Water	0.03 ± 0.00

### Scale-up of enzymatic hydrolysis

Enzymatic hydrolysis at high solids loadings is another key to scale-up of lignocellulosic biochemical conversion processes, because of potentially higher sugar and ethanol titers and low hydraulic loads. However, high solids loading can pose rheological challenges, reduce mass and heat transfer efficiency, and increase the concentration of enzyme inhibitors in the system, resulting in lower conversion of glucan and xylan into fermentable sugars. Previous reports have simulated different operational scenarios and compared them in terms of productivity, yield, and numbers of stages and unit operation for materials from steam explosion, dilute acid, alkali pretreatment processes [19,37-41]. So far, there is limited information on large-scale enzymatic hydrolysis of IL pretreated material, especially at high solids loading as most of the saccharification data were obtained at laboratory scales with 1-2% loading of IL pretreated materials [16-18], which cannot be directly translated to industrially relevant scales. Thus, focused scale-up studies at high solid loadings are required to fit in the intermediate scale levels in the current study.

**Figure 6:**
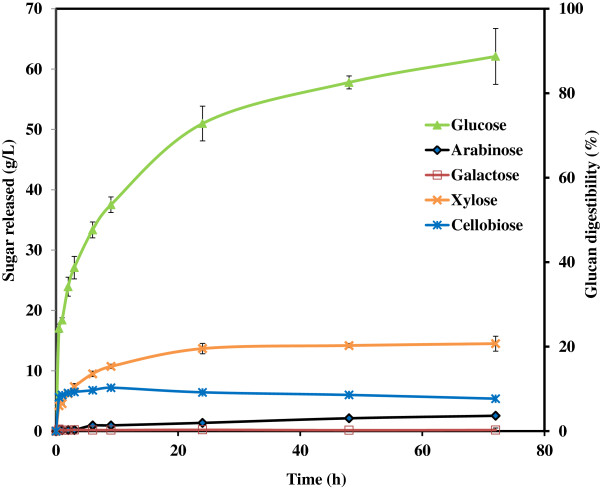
**Sugar released from 2L scale enzymatic hydrolysis of [C**_**2**_**mim][OAc] pretreated switchgrass.**

Figure 6 shows the typical fermentable sugar profile for a 72 h enzymatic saccharification of pretreated switchgrass at 10% solid loading in a 2 L reactor with 1.5 L working volume. It is observed that the concentrations of glucose, xylose and cellobiose increase rapidly in the first 30 minutes. The glucose concentration then continues to rapidly increase, whereas the xylose concentration follows a fairly steady increase until about 24 h, and then a very slow, almost negligible increase up to 72 h. The cellobiose level reached a plateau at 2 h and started to decrease after about 24 h, indicating that the β-glucosidases are highly active and are not being inhibited by product formation. The rapid initial increase for all three sugar concentrations coincided with a significant reduction in biomass solubilization and slurry viscosity. This is attributed to the highly disrupted cellulose structure, increased porosity and accessibility in the IL pretreated fiber [7]. Furthermore, the easily solubilized xylo-oligosaccharides generated from the pretreated switchgrass promoted rapid xylose release, whereas the second stage of xylan conversion, from approximately 3 h to 24 h reflects the hydrolysis of xylan polymers in the biomass. Unfortunately, this process essentially leveled off after 24 h with little further production of xylose in spite of the fact that only 65.0% of available xylan in the pretreated solids had been hydrolyzed.

The evolution of glucose is more sustained, proceeding at a very fast kinetic rate from 1 h to 24 h, and then at a relatively lower rate from 24 to 72 h, at which point 99.8% of available glucan in the IL pretreated solids has been hydrolyzed to glucose. High contents of glucose at 62.1 g/L combined with 5.4 g/L of cellobiose demonstrate high titers of sugar concentration. The glucan digestibility is consistent with previous reports demonstrating the equal effectiveness of [C_2_mim][OAc] pretreatment at 3% loading and saccharification at 15% loading at small scales of 5 to 25 mL [16,18], suggesting a successful 60 fold scale up. However, the xylan digestibility was lower than that reported for small-scale studies, suggesting the following possibilities: 1) enzymes in the HTec2 cocktail may be deactivated/inhibited by residual [C_2_mim][OAc] or end-product accumulation at high biomass loading, 2) [C_2_mim][OAc] pretreatment at larger scales produces xylan that is less accessible to enzymes than the xylan produced at smaller scales. Thus further optimization of the enzyme cocktail may be required to obtain maximal sugar production.

### Mass balance and energy flow

**Figure 7:**
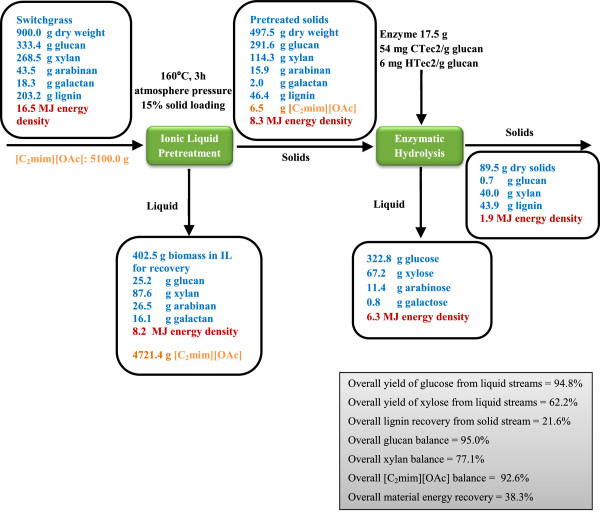
**Mass balance and energy flow during [C**_**2**_**mim][OAc] pretreatment and subsequent enzymatic hydrolysis.**

An analysis of the mass balance and energy flow of the ionic liquid pretreatment, the subsequent enzymatic saccharification, and their resultant composition of the products generated is summarized in Figure 7 to develop a clear overview of the technology scale up. On the 900 g basis of untreated switchgrass, 497.5 g of pretreated solids were recovered that retain 87.5% of glucan, 42.6% of xylan and 22.8% of lignin. On the same basis, 25.2 g of glucose olignomers, 87.6 g of xylo-oligomers, plus 26.5 arabinan and 16.1 g of galactan, respectively, were recovered post hydrolysis. Approximately 322.8 g of glucose, 67.2 of xylose and 11.4 g of arabinose, respectively, were recovered from enzymatic hydrolysis of the recovered solids. Furthermore, of the 5100 g of ionic liquid used, 4721 g was detected in the liquid fraction and available for further recovery and recycling. A small portion of IL (6.5 g) was retained with pretreated switchgrass, and potentially posed inhibitory effects for the subsequent enzymatic hydrolysis and microbial fermentation. The overall IL balance closure is higher than 92%.

The material balance indicates some mass loss during pretreatment and enzymatic hydrolysis, yet the overall glucose recovery in the liquid stream remains over 94%, confirming that IL pretreatment can preserve most of the sugars and substantially enhance the effectiveness of enzymatic hydrolysis. The overall glucan balance closure on the basis of the recovered solids after pretreatment (95.0%) is higher than xylan (77.1%), which is attributed to the greater chemical robustness of glucose during the IL pretreatment [17]. A fraction of the hemicellulose remained in the liquid stream after pretreatment as well as in the residual solid after saccharification but is not lost to the overall conversion process [35]. During pretreatment, a significant amount of lignin was also solubilized into the liquid stream, causing the lignin reduction in the pretreated solids. However, the residual solids after enzymatic saccharification are also rich in lignin content, indicating potential opportunities for lignin valorization. For a long-term development of biorefinery, lignin supply will progressively increase as many types of lignocellulosic feedstocks are implemented in the future. Adding value to the lignin rich residue will significantly enhance the competitiveness of biomass-to-biofuels conversion [42].

One of the considerations that must be taken into account is the energy density of the feedstock itself. Energy density plays an important role in the overall energy and cost balance of the biofuel production process. A biomass feedstock with low energy density is less energy efficient to convert into a biofuel than one with a higher energy density due to the relatively high energy required for transportation, storage and distribution of the feedstock from the field to the biorefinery gate [17,43,44]. Lignin contains the higher energy content than cellulose and hemicellulose, and can be combusted to provide heat and/or power for the biorefinary process, and provide some excess power to generate additional revenue [45,46]. To understand how the IL pretreatment affects lignin recovery and energy flow, lignin content and energy density analysis of untreated, pretreated and saccharified switchgrass were conducted and are presented in Table 2. Results clearly show that, compared with 22.6% in untreated material, the lignin content was significantly decreased to 9.33% after IL pretreatment, however, after enzymatic hydrolysis, the residual solids are rich in lignin (49.08%) due to the efficient conversion of polysaccharides into soluble sugars. Correspondingly, energy density of untreated (18.37 KJ/g), pretreated (16.62 KJ/g) and saccharified switchgrass (21.72 KJ/g) tracks well with their respective lignin content. Details about the energy density analysis and the relative correlation with three major components in various feedstocks are currently under investigation. The overall lignin recovery from solid stream is 21.6%, indicating a large amount of lignin has dissolved in IL upon pretreatment and requires further separation. The material energy flow is also summarized in Figure 7 based on the energy density data from Table 2, and the overall recovery is 38.3% with high energy contents left in liquid stream, suggesting further energy recovery is highly warranted.

**Table 2:** Lignin content and energy density in three types of starting and recovered solids

**Biomass solids**	**Lignin (%)**	**Energy density (KJ/g)**
Untreated	22.58 ± 1.01	18.37 ± 0.03
Pretreated	9.33 ± 0.38	16.62 ± 0.74
Saccharified	49.08 ± 1.46	21.72 ± 0.95

It should be addressed that due to their current high cost, recovery and recycle of ILs has been given more and more attentions for the requirements of commercial use in biomass pretreatment. These include using anti-solvent such as acetone followed by distillation/evaporation for separation [9,47], biphasic system with addition of an aqueous solution containing kosmotropic anion, such as phosphate, carbonate or sulfate [48,49], and sequential membrane filtration and vacuum evaporation post sugar extraction from aqueous IL hydrolysate. Although these results show that separation and recovery of IL can be achieved by various methods, to date, all these potential alternatives have been limited to the lab-scale level of development and require more investigation before scale-up can occur. The process employed in the current report doesn’t involve IL recovery, optimization for washing method, separation method, and removal of the remaining water, however, as a continuation of this study, JBEI and ABPDU are working together on the scale up demonstration of newly developed wash-free IL processes [26,35] and liquid-liquid extraction and filtration based IL recycle technologies. The present work provides an essential first step to understand and evaluate the scale up effect and important parameters, and requires to be further developed for a commercially scalable and cost competitive process.

## Conclusions

To the best of our knowledge, this is the first demonstration in the scientific literature for the scale-up of IL pretreatment by 600-fold and subsequent enzymatic saccharification by 60-fold at solid loadings up to 15% (w/w). The results generated are consistent with those from the small-scale experiments that have been conducted at JBEI and elsewhere, and indicate there are no fundamental issues in terms of performance associated with the scale-up of an IL-based conversion technology. High solids loading during pretreatment and enzymatic hydrolysis offers several industrial advantages, including decreased reactor size, increased sugar titer and hydrolysis rate, and decreased water consumptions. The results also provide clear evidence of enzymatic inhibition, which is magnified by the fact that pretreated materials were not washed completely prior to hydrolysis, and thus, the inhibitors generated during pretreatment and washing will persist into hydrolysis and the downstream steps. Thus, the optimization and engineering of the IL tolerant enzyme cocktail and scale up design/operation of more effective IL pretreatment and post separation system must be realized before a commercially viable process is realized. The knowledge gained from this initial scale-up study is an essential first step in demonstrating the commercial viability of this promising pretreatment technology.

## Materials and methods

### Raw materials

Switchgrass (*Panicum virgatum*) was kindly provided by Dr Putnam’s lab at University of California, Davis. The samples were air dried first until the moisture was less than 10%, and then grounded with Thomas Wiley Mill fitted with a 2 mm screen (Model 4, Arthur H. Thomas Co., Philadelphia, PA, USA). The samples were stored at 4°C cold room for use in all experimentation. The particle size distribution of this material after grinding was determined by following ASTM D1511-10 using a sieve shaker (Vibratory Sieve Shaker AS 200, Retsch, Newtown, PA, USA) and the biomass materials passing sieves of specific mesh sizes was measured (ASTM International, 2010). The majority of the materials by weight (54%) have particle sizes ranging from 0.1 to 0.6 mm. Cellulase (Cellic® CTec 2) and hemicellulase (Cellic® HTec2) were generously provided by Novozymes (Davis, CA). 1-Ethyl-3-methyl-limidazolium acetate ([C_2_mim][OAc]), BASF, purity ≥90%) was used as the ionic liquid for the pretreatment experiments. Trifluoroacetic acid (TFA), ethanol, acetic acid, sodium acetate, sulfuric acid, sodium hydroxide, and the monosaccharides including arabinose, galactose, xylose, glucose, and cellobiose were purchased from Sigma-Aldrich (St. Louis, MO).

### Reactor setup and operation for large scale IL pretreatment

Optimal pretreatment temperature (160°C) and residence time (3 h) using ionic liquid and switchgrass for fermentable sugar production were chosen from previous small scale studies [7,15]. In this scale up study, 15% (w/w) biomass/IL solution was prepared by combining 900 g (dry basis) of switchgrass with 5.1 kg [C_2_mim][OAc] in the 10 L Parr reactor (Model 4558, Parr Instrument Company, Moline, Illinois, USA) in triplicates. For each run, the reactor was sealed and the reactants were heated at 160°C for 3 h with a stirring speed of 50 rpm from an anchor impellor. Temperature ramping (to 160°C) and cooling (to 60°C) times were approximately 30 and 20 min, respectively. After 3 h incubation, the reactor was cooled down to 60°C with chilled water through the cooling coils inside the reactor. The reactor was open to sample 25 gram of biomass/IL slurry for viscosity measurement, and then 6 L of preheated hot water (60°C) was slowly pumped into the reactor, causing precipitation of the biomass into large chunks. Both solids and liquids were transferred into a 50 L bucket for overnight soaking. This was followed by a 2-min homogenization step to obtain the uniform dispersion of small particles in the solutions with a laboratory blender (LBC 15, Waring Laboratory, Torrington, CT).

### Biomass washing

The homogenized biomass was first separated from the IL-rich pretreatment liquid by filtration through cheese cloth (100% natural bleached cotton fiber) at 25°C [50]. Then the recovered solids were washed and dewatered through cheesecloth to remove IL and dissolved byproducts such as lignin and hemicellulose that might inhibit enzymes in the subsequent enzymatic hydrolysis. The washing steps included 3 water washes (12 L), 1 ethanol wash (6 L), 2 ethanol/water (1:1, v/v, 6 L) washes, and 1 final water wash (12 L) at room temperature. Each time water or ethanol was squeezed extensively from the biomass through cheesecloth [50]. All water wash effluent were collected separately and stored for further analysis of IL concentration and soluble sugar contents. The washed solids were collected to determine moisture content and calculate the solid recovery from IL pretreatment. About 10 g (dry basis) of the pretreated biomass was dried in the vacuum oven at 45°C to constant weight for composition and energy density analysis, and the rest was stored in sealed containers at 4°C for enzymatic hydrolysis.

### Chemical characterization of switchgrass

Acid-insoluble lignin, and structural carbohydrates, i.e., glucan, xylan, arabinan and galactan, of switchgrass before and after pretreatment were determined according to analytical procedure of the National Renewable Energy Laboratory (NREL) by a two-step sulfuric acid hydrolysis [51,52]. Carbohydrates were analyzed by high performance anion exchange chromatography (HPAEC) on an ICS-3000 system (Dionex, Sunnyvale, CA), equipped with an electrochemical detector and a 4 × 250 mm CarboPac PA20 analytical column. Elution was initiated with 97.2% (v/v) water and 2.8% (v/v) 1 M NaOH for first 15 min, with 20 μL injection volume. Elute concentration was then switched to 55.0% (v/v) water and 45.0% (v/v) 1 M NaOH for next 20 min and returned to 97.2% (v/v) water and 2.8% (v/v) 1 M NaOH for the last 10 min to equilibrate the column. The flow rate was 0.5 mL/min. The monosaccharides including arabinose, galactose, xylose and glucose, were used as the external standards for HPAEC and prepared at levels of 0 to 100 mM before use. Absorbance reading of acid soluble lignin was taken at 205 nm using a UV–Vis spectrophotometer (Shimadzu UV-2401) with high purity quartz cuvettes with a 1 cm pathlength. The extinction coefficient of 110 L/g cm was used for the calculation of acid soluble lignin for switchgrass [53].

To obtain the sugar balance from dissolution of hemicelluloses during IL pretreatment, a TFA hydrolysis was performed. The supernatants from the water wash steps were collected and concentrated, 30 μL of solution was diluted 10-fold with water and treated with 150 μL of TFA at 120°C for 1 h. The hydrolyzed solution was centrifuged at 10,000 g for 10 min, and the supernatant was analyzed using HPAEC for the monosaccharide analysis after evaporation of the TFA residues in a Centri-Vap Vacuum Concentrator (Labconco Corp, MO) at 30°C overnight.

### Large scale enzymatic saccharification

To investigate the washing efficiency on the IL removal in the recovered solids and the effect of IL content on enzymatic saccharification, a small scale study was first conducted by hydrolyzing the solid samples recovered from each washing step. The experiments were run at 2% (w/w) in duplicate at 50°C and 150 rpm in a reciprocating shaker. The total batch volume was 25 mL with cellulase (CTec2) concentration of 54 mg protein/g glucan and endoxylanase (HTec2) concentration of 6 mg protein/g glucan to ensure fast hydrolysis. The protein concentrations of the two commercial enzyme mixtures (CTec2 190 g/L, HTec2 174 g/L) were determined by the Bradford assay (Bio-Rad, Hercules, CA) using bovine serum albumin as a standard.

After 72 h, the hydrolysates were analyzed on HPAEC for the monosaccharides release and their correlation with IL contents. For the scale up experiment of enzymatic hydrolysis, the IL pretreated switchgrass samples from final washing step were fed into 2 L IKA reactor (IKA LR-2.ST, IKA Works, Wilmington, NC) in triplicates. The samples were kept at 10% (w/w) biomass loading in water with a working volume of 1.5 L and continuously mixed at 150 rpm for enzymatic hydrolysis using an anchor impeller. The Novozymes cocktails were added into each reactor with cellulase (CTec2) concentration of 54 mg protein/g glucan and endoxylanase (HTec2) concentration of 6 mg protein/g glucan. The reaction was monitored by taking samples from 0, 0.5,1,2,3, 6, 9, 24, 48 and 72 h of time intervals and measuring monomeric sugar production. After the hydrolysis time elapsed, the hydrolysates were immediately transferred into the plastic containers and placed at 4°C to avoid further reaction.

### IL measurement

The C_2_mim^+^ content was determined using a Dionex UltiMate 3000 UHPLC (Dionex, Sunnyvale, CA) with UV–vis detecter at 240 nm wavelength. A Dionex acclaim 120 C18 column achieved the separation by isocratic elution with a mobile phase consisting of 20 mM ammonium acetate and 1% acetic acid at 1.0 mL/min and 20°C. The IL supernatant taken from various washing steps were directly measured for IL content after proper dilutions. To measure the residual [C_2_mim][OAc] remaining in the biomass after various wash steps, the recovered solids were saccharified for 120 hours using an extreme excess of enzymes (540 mg/g glucan of CTec2, 300 mg/g glucan of HTec2) at 50°C and pH 5.5 to completely solubilize the solids and ensure the [C_2_mim][OAc] attached to the solids went into the liquid fraction. Then the liquid samples were diluted and measured on HPLC for [C_2_mim][OAc] content.

### Viscosity measurement

The rheological properties of switchgrass-IL slurry were investigated for samples before pretreatment, after pretreatment, and after pretreatment and washing (removal of IL) using stress controlled Malvern Kinexus Rheometer (Worcestershire, UK) a with 40 mm diameter parallel plate geometry. A gap height of 5 mm was used and care was taken to avoid air bubbles being trapped in the sample by obtaining a bulge of the sample at the edges of the plate. Oscillatory stress sweeps were conducted at 25°C from 0.01 to 1000 Pa under a constant frequency of 5 Hz. A clear viscoelastic region was observed between 1 and 100 Pa. Accordingly, an oscillatory frequency sweep was conducted at 25°C between 0.01 to 150 Hz at a constant stress of 10 Pa. Viscoelastic properties including elastic modulus (G’, Pa), viscous modulus (G”, Pa), and complex viscosity (η*, Pa-s) were measured and reported in this study.

### Energy density measurement

The solid samples before and after IL pretreatment were subjected to a standard bomb calorimeter (C2000 Oxygen Bomb Calorimeter, IKA Works, Wilmington, NC) for energy density measurement. All samples were dried in a vacuum oven at 40°C until the moisture content is lower than 4%, ball milled to pass a 20 mesh screen and then compressed into pellets using a hydraulic pelletizer (MTI 12 T pelletizer, MTI, Richmond, CA) prior to being weighed. Heat content was determined by burning the samples with excess of oxygen at a pressure of 435 psi in a sealed steel bomb, which is regarded as a near-adiabatic system.

## Abbreviations

IL: Ionic liquid; JBEI: Joint bioenergy institute; ABPDU: Advanced biofuels process demonstration unit; [C2mim][OAc]: 1-ethyl-3-methylimidaolzium acetate; HPAEC: High performance anion exchange chromatography; TFA: Trifluoroacetic acid; NREL: National renewable energy laboratory.

## Competing interests

The authors declare that they have no competing interests.

## Authors’ contributions

CL and DT conducted the pretreatment and saccharification experiments. CL conducted data analysis and drafted the manuscript. DT carried out the viscosity measurement and contributed to the viscosity related part of the manuscript. WH and JLG performed the energy density and IL measurement. JW performed the compositional and hydrolysate analysis. SS, BAS and KS contributed to the original experimental design and modifications of the draft. SS, BAS, JLG and KS coordinated and supervised the collaboration project. All authors read and approved the final manuscript.
